# A high incidence of vertebral fracture in women with breast cancer

**DOI:** 10.1038/sj.bjc.6690188

**Published:** 1999-03

**Authors:** J A Kanis, E V McCloskey, T Powles, A H G Paterson, S Ashley, T Spector

**Affiliations:** WHO Collaborating Centre for Metabolic Bone Diseases, University of Sheffield Medical School, Beech Hill Road, Sheffield S10 2RX, UK; Breast Unit and Computer Department, Royal Marsden Hospital, Downs Road, Sutton, Surrey, SM2 5PT, UK; Department of Medicine, Tom Baker Cancer Centre, 1331–29 Street NW, Calgary, Alberta, T2N 4N2, Canada; Twin Research and Osteoporosis Unit, St Thomas' Hospital, London, SE1 7EH, UK

**Keywords:** vertebral fracture, breast cancer, skeletal metastases

## Abstract

Because treatment for breast cancer may adversely affect skeletal metabolism, we investigated vertebral fracture risk in women with non-metastatic breast cancer. The prevalence of vertebral fracture was similar in women at the time of first diagnosis to that in an age-matched sample of the general population. The incidence of vertebral fracture, however, was nearly five times greater than normal in women from the time of first diagnosis [odds ratio (OR), 4.7; 95% confidence interval (95% CI), 2.3–9.9], and 20-fold higher in women with soft-tissue metastases without evidence of skeletal metastases (OR, 22.7; 95% CI, 9.1–57.1). We conclude that vertebral fracture risk is markedly increased in women with breast cancer. © 1999 Cancer Research Campaign


British Journal of Cancer (1999) 79(7/8), 1179–1181
© 1999 Cancer Research Campaign
Article no. bjoc.1998.0188

### A high incidence of vertebral fracture in women with breast cancer

**JA Kanis[1], EV McCloskey[1], T Powles[2], AHG Paterson[3], S Ashley[2] and T Spector[4]**

[1]WHO Collaborating Centre for Metabolic Bone Diseases, University of Sheffield Medical School, Beech Hill Road, Sheffield S10 2RX, UK; [2]Breast Unit and Computer Department, Royal Marsden Hospital, Downs Road, Sutton, Surrey SM2 5PT, UK; [3]Department of Medicine, Tom Baker Cancer Centre, 1331–29 Street NW, Calgary, Alberta T2N 4N2, Canada; [4]Twin Research and Osteoporosis Unit, St Thomas' Hospital, London SE1 7EH, UK

**Summary** Because treatment for breast cancer may adversely affect skeletal metabolism, we investigated vertebral fracture risk in women with non-metastatic breast cancer. The prevalence of vertebral fracture was similar in women at the time of first diagnosis to that in an age-matched sample of the general population. The incidence of vertebral fracture, however, was nearly five times greater than normal in women from the time of first diagnosis [odds ratio (OR), 4.7; 95% confidence interval (95% CI), 2.3–9.9], and 20-fold higher in women with soft-tissue metastases without evidence of skeletal metastases (OR, 22.7; 95% CI, 9.1–57.1). We conclude that vertebral fracture risk is markedly increased in women with breast cancer.

**Keywords:** vertebral fracture; breast cancer; skeletal metastases

Metastases from breast cancer appear to have a predilection for the skeleton. In addition to focal skeletal disease, there is evidence that breast cancer is associated with generalized disturbances in skeletal metabolism.

Increased resorption and accelerated turnover of bone have been shown in biopsies taken at sites distant from skeletal metastases (Taube et al, 1994), perhaps mediated by the systemic secretion of parathyroid hormone-related protein (PTHrP) which is expressed in breast cancer tissue (Orloff et al, 1989; Powell et al, 1991).

In addition to the expression of PTHrP, both chemotherapy and a chemotherapy-induced menopause are likely to increase the risk of osteoporosis (Rivkees and Crawford, 1988; Saarto et al, 1997). Whereas tamoxifen may protect against bone loss in post-menopausal women, recent evidence suggests that bone losses may be accelerated in women with normal ovarian function (Powles et al, 1996; Saarto et al, 1997). For these reasons, we wished to determine whether the risk of osteoporotic fractures was higher in women with non-metastatic breast cancer than in the healthy population.

### PATIENTS AND METHODS

We studied two populations of women with breast cancer and a control population of women randomly drawn from the female population. Eighty-two women had histologically proven breast cancer and recurrent disease diagnosed according to standard criteria (Breast Cancer Task Force, 1977; Hayward et al, 1978), but without skeletal metastases as judged by skeletal radiographs and scintigraphy. They comprised a cohort of women in whom the effects of clodronate on the incidence of skeletal metastases and associated morbidity were studied. The findings of this trial have been published elsewhere (Kanis et al, 1996).

*Received 26 January 1998*
*Revised 26 June 1998*
*Accepted 30 July 1998*

*Correspondence to:* JA Kanis

After initial assessment, patients were randomized to receive clodronate by mouth, 1600 mg daily as four capsules, or an identical placebo supplied by Leiras Oy, Helsinki, Finland. Patients were instructed to take four capsules daily in the morning 1 h before meals and away from calcium-containing liquids. Bone scintigraphy and skeletal radiographs (hands, pelvis, skull, lateral lumbar and thoracic spine) were obtained at 6-monthly intervals.

The second population of women with breast cancer comprised 352 women recruited at the time of the first diagnosis to study the effects of clodronate, 1600 mg daily by mouth, on the incidence of skeletal metastases. None of the women had radiographic or scintigraphic evidence of skeletal disease. No selection was made on the basis of histological grade or the involvement of axillary nodes. After diagnosis, 36% of women received tamoxifen, 18% chemotherapy and 43% both chemotherapy and tamoxifen. After randomization to clodronate or placebo treatment, patients were assessed at 6-monthly intervals and spinal radiographs taken yearly. The study is continuing and remains double blind, so that the data are presented irrespective of treatment allocation.

A control population comprised 776 women aged 45–69 years randomly drawn from a catchment population in London (Spector et al, 1993). At initial assessment, 193 women (25%) were current or past users of hormone replacement treatment (HRT). The prevalence of vertebral fracture was similar in women exposed to HRT (3–6%) to that in non-users (5–7%) and the groups were combined for analysis. All women had a radiographic assessment of the spine which was repeated 3 years after the initial assessment.

Anteroposterior and lateral radiographs of the thoracic spine (taken during inspiration) and the lumbar spine were obtained at a standard target to film distance of 105 cm, with the thoracic and lumbar film centred on T7 and L3 respectively. Radiographs were read blindly at a single centre. The anterior, posterior and central heights of each vertebra from T4 to L5 were reported using a semiautomated technique and vertebral fractures defined using an algorithm previously established (McCloskey et al, 1993). In addition, the severity of vertebral fractures was graded according to the degree of deformity. A central compression fracture was assigned

**Table 1** Characteristics of controls and women with breast cancer at study entry

| | Study group | | | | | |
|---|---|---|---|---|---|---|
| | Controls | | Breast cancer at diagnosis | | Soft-tissue relapse | |
| | Mean | s.d. | Mean | s.d. | Mean | s.d. |
| Age (years) | 54.1 | 6.0 | 54.5 | 10.4 | 59.4 | 10.2 |
| Height (cm) | 161.8 | 6.2 | 162.5 | 6.3 | 162.2 | 6.0 |
| Weight (kg) | 66.7 | 11.6 | 67.8 | 12.0 | 63.2 | 10.0 |
| BMI (kg $m^{-2}$) | 25.4 | 4.1 | 25.6 | 4.3 | 23.9 | 4.1 |
| Follow-up (years) | 2.9 | 0.3 | 2.1 | 1.2 | 1.8 | 1.4 |

**Table 2** Prevalence (%) of vertebral fractures

| Age (years) | Controls (%) | Breast cancer at diagnosis (%) | Breast cancer at soft-tissue relapse (%) |
|---|---|---|---|
| <50 | 2.7 | 4.0 | 40.0 |
| 50–59 | 5.9 | 7.4 | 17.6 |
| 60–69 | 6.7 | 4.8 | 30.0 |
| 70+ | – | 13.0 | 33.0 |
| All ages | 5.2 | 6.0 | 30.5 |
| <70 | 5.2 | 5.5 | 25.0 |
| Mean number of fractures | 1.4 | 1.2 | 1.8 |

a score of 1, an anterior wedge fracture a score of 2, and a complete collapse (crush fracture) a score of 3.

### RESULTS

The characteristics of the three populations are shown in Table 1. There were no differences in mean age, height or weight between the control population and women with newly diagnosed breast cancer, but patients with recurrent breast cancer were significantly older than controls. Both the crude and age-adjusted prevalence of vertebral fractures was similar in women with cancer at first diagnosis to that in the control population, but was more than sixfold higher in women with soft-tissue relapse ($\chi^2$ = 73.2; $P < 0.0001$; Table 2). In those women with vertebral fracture, there was no difference in the average number of fractures between the population with newly diagnosed breast cancer and the population controls (1.2 vs 1.4 fractures per patient respectively). The mean number of fractures in women with recurrent breast cancer was higher than in other groups (1.8), but the difference was not statistically significant.

In contrast to data on prevalence, the incidence of vertebral fracture was markedly increased in women followed from diagnosis of breast cancer compared with normal controls. Fractures occurred in 1.5% of controls and 5.4% of breast cancer patients followed from diagnosis, despite a shorter duration of follow-up in the latter (2.9 vs 2.1 years respectively). Incidence was higher in those women with breast cancer irrespective of age (Table 3), and was even higher in women with soft-tissue relapse but without skeletal metastases. The age-adjusted risk of vertebral fracture was higher in women with a prevalent fracture at study entry [odds ratio (OR), 3.4; 95% confidence interval (95% CI), 1.5–7.8]. When fracture risk was adjusted for age, duration of follow-up and prevalent fracture, the risk was 4.7 (95% CI, 2.3–9.9; $P < 0.0001$) in women newly diagnosed with breast cancer and 22.7 (95% CI, 9.1–57.1; $P<0.0001$) in the women with recurrent breast cancer. In women with vertebral fracture, the mean number of vertebral fractures was significantly greater in women with soft-tissue metastases than in controls ($P<0.05$), and the severity of fractures was significantly greater in these women than in controls or in women with newly diagnosed breast cancer.

Several patients developed vertebral fractures in association with skeletal metastases. In women followed from diagnosis, 25 (7%) developed skeletal metastases, and in women with recurrent breast cancer 28 (34%) developed skeletal metastases. Because vertebral fractures may have resulted from metastatic

**Table 3** Annual incidence (%) and severity (± s.e.m.) of vertebral fractures

| Age (years) | Controls (%) | Breast cancer at diagnosis (%) | Breast cancer at soft-tissue relapse (%) |
|---|---|---|---|
| <50 | 0.45 | 1.87 | 15.38 |
| 50–59 | 0.25 | 2.68 | 12.00 |
| 60–69 | 1.06 | 2.87 | 14.63 |
| 70+ | – | 5.26 | 28.57 |
| All ages | 0.53 | 2.72 | 19.21 |
| <70 years | 0.53 | 2.64 | 13.46 |
| >50 years | 0.55 | 2.90 | 16.07 |
| Mean number of fractures[a] | 1.08 ± 0.29 | 1.45 ± 0.94 | 1.69 ± 1.04 |
| Mean severity score[a] | 2.00 ± 1.04 | 2.45 ± 1.82 | 4.10 ± 2.51 |

[a]In patients with fracture

*© Cancer Research Campaign 1999*

disease, incidence rates were recalculated excluding patients with skeletal metastases. The incidence of vertebral fractures remained significantly higher than controls in women followed from diagnosis (2%, OR, 2.8; 95% CI, 1.3–6.2) and in women with soft-tissue relapse (16.5%; OR, 24.5; 95% CI, 10.7–55.9).

### DISCUSSION

The principal finding in this study was the high incidence of vertebral fracture in women with breast cancer, but without clinical evidence for skeletal metastases. In women at the time of first diagnosis of breast cancer, the incidence of vertebral fracture over the next 3 years was nearly fivefold higher than in the normal population. The risks were more than 20-fold greater in women with recurrent breast cancer but no evidence of skeletal metastases. The adequacy of the control population is suggested by the observation that the prevalence of vertebral fracture was similar in women at first diagnosis to that in women drawn randomly from the general population. A previous study from Rochester showed comparable findings (Utz et al, 1987). The same study also showed an increase in risk of vertebral fractures in women with breast cancer, but these were attributed to skeletal metastases.

It is likely that the risk of vertebral fracture is underestimated because half of the women with breast cancer were receiving clodronate, which has been shown to decrease the risk of vertebral fracture both in breast cancer (Paterson et al, 1993; Kanis et al, 1996) and in osteoporosis (Filipponi et al, 1996). Clodronate also decreases bone loss in women at the time of diagnosis of breast cancer (Powles et al, 1997; Saarto et al, 1997). If clodronate decreased the risk of fractures by about 50%, as suggested by these studies, then the risks we found would be underestimated by 25%.

As mentioned, the prevalence of fracture in women with breast cancer at the time of first diagnosis was similar to that in control women, suggesting that the increase in risk during follow-up depended upon factors operating subsequent to diagnosis. The prevalence and the risk was much higher in women with soft-tissue recurrence at a later stage of the disease, which is consistent with this view. There were small differences in the duration of follow-up between groups, but adjustment for these made no difference to the risks.

It is unlikely that high rates of vertebral fracture were due to missed skeletal metastases because the majority did not develop spinal metastases during the conduct of the study. Moreover, as mentioned previously, there are several reasons why bone losses should be excessive in such patients. These considerations suggest that vertebral fractures are related to osteoporosis. The degree of risk is sufficiently high that intervention can be cost-effectively targeted to women at the time of diagnosis of breast cancer (Kanis et al, 1997). It will, however, be important to determine to what extent alterations of menopausal status and the use of concurrent medication affect bone loss, which because of the small sample size was not possible in the present study.

### ACKNOWLEDGEMENTS

The studies on clodronate were supported by Leiras Oy. We are grateful to the staff and patients of Chingford Hospital and Handsworth Avenue Practice.

### REFERENCES

Breast Cancer Task Force. Treatment Committee (1977) National Cancer Institute, Breast Cancer. Suggested protocol guidelines for combination chemotherapy trials and for combined modality trials. Washington, DC: US Department of Health, Education and Welfare. Publication no. (NIH) 77–1192

Filipponi P, Christallini S, Rizzello E, Policani G, Fedeli L, Gregorio F, et al (1996) Cyclical intravenous clodronate in postmenopausal osteoporosis. Results of a long-term trial. *Bone* **18**: 179–184

Hayward JL, Meakin JW and Stewart HJ (1978) Assessment of response and recurrence in breast cancer. *Semin Oncol* **5**: 445–449

Kanis JA, Powles T, Paterson AHG, McCloskey EV and Ashley S (1996) Clodronate decreases the frequency of skeletal metastases in women with breast cancer. *Bone* **19**: 663–667

Kanis JA, Delmas P, Burckhardt P, Cooper C and Torgerson D, on behalf of the EFFO (1997) Guidelines for the diagnosis and management of osteoporosis. *Osteoporosis Int* **7**: 390–406

McCloskey EV, Spector TD, Eyres KS, Fern DE, O'Rourke N, Vasikaran S and Kanis JA (1993) The assessment of vertebral deformity – a method for use in population studies and clinical trials. *Osteoporosis Int* **3**: 138–147

Orloff JJ, Wu TL and Stewart AF (1989) Parathyroid hormone-like proteins: biochemical responses and receptor interactions. *Endocrinol Rev* **10**: 476–495

Paterson AHG, Powles TJ, Kanis JA, McCloskey E, Hanson J and Ashley S (1993) Double-blind controlled trial of oral clodronate in patients with bone metastases from breast cancer. *J Clin Oncol* **11**: 59–65

Powell GJ, Southby J, Danks JA, Stillwell RG, Hayman JA, Henderson MA, et al (1991) Localisation of parathyroid hormone-related protein in breast cancer metastases: increased incidence in bone compared with other sites. *Cancer Res* **51**: 3059–3061

Powles TJ, Hickish T, Kanis JA, Tidy A and Ashley S (1996) The effect of tamoxifen on lumbar bone mineral density in pre- and postmenopausal women. *J Clin Oncol* **14**: 78–84

Powles TJ, McCloskey E, Paterson AHG, Ashley S, Tidy A and Kanis JA (1997) Oral clodronate reduces the loss of bone mineral density in women with operable primary breast cancer. *J Clin Oncol*, submitted for publication, ASBMR abstract

Rivkees AS and Crawford JD (1988) The relationship of gonadal activity and chemotherapy-induced gonadal damage. *JAMA* **259**: 2123–2125

Saarto T, Blomqvist C, Valimaki M, Makelo P, Sarna S and Elomaa I (1997) Chemical castration induced by adjuvant cyclophosphamide, methotrexate and fluorouracil chemotherapy causes rapid bone loss that is reduced by clodronate: a randomised study in premenopausal breast cancer patients. *J Clin Oncol* **15**: 1341–1347

Spector TD, McCloskey E, Doyle DV and Kanis JA (1993) Prevalence of vertebral fractures in women and the relationship with bone density and symptoms. The Chingford study. *J Bone Miner Res* **8**: 817–822

Taube T, Elomaa I, Blomqvist C, Beneton MNC and Kanis JA (1994) Histomorphometric evidence for osteoclast mediated bone resorption in metastatic breast cancer. *Bone* **15**: 161–166

Utz JP, Melton LJ, Kan SH and Riggs BL (1987) Risk of osteoporotic fractures in women with breast cancer: a population-based cohort study. *J Chron Dis* **40**: 105–113

© Cancer Research Campaign 1999
